# Sensory Thresholds and Peripheral Nerve Responses in Chronic Tension-Type Headache and Neuropsychological Correlation

**DOI:** 10.3390/jcm11071905

**Published:** 2022-03-29

**Authors:** Rosalinda Romero-Godoy, Sara Raquel Romero-Godoy, Manuel Romero-Acebal, Mario Gutiérrez-Bedmar

**Affiliations:** 1 Department of Nursing and Physiotherapy, University of Balearic Islands, 07122 Palma, Spain; 2 Cognitive Affective Neuroscience & Clinical Psychology Research Group, Institute of Health Science Research (IUNICS-IdISBa), ECYCS Research Group, University of Balearic Islands, 07120 Palma, Spain; 3 Neurology Department, Virgen de la Victoria University Hospital, 29010 Malaga, Spain; sararaquelrogo@gmail.com (S.R.R.-G.); mromeroa@uma.es (M.R.-A.); 4 Preventive Medicine and Public Health Department, School of Medicine, University of Malaga, 29010 Malaga, Spain; 5 Biomedical Research Institute of Malaga (IBIMA), 29010 Malaga, Spain; 6 CIBERCV Cardiovascular Diseases, Carlos III Health Institute, 28029 Madrid, Spain

**Keywords:** chronic tension-type headache, electrical nerve stimulation, sensory threshold, pain threshold, central sensitization, peripheral sensitization, Aβ fiber excitability

## Abstract

Chronic tension-type headache (CTTH) is a common disease with no fully defined pathophysiological processes. We designed a study to value electrophysiological responses in these patients and their correlation with possible psychopathological manifestations in order to deepen understanding of central and peripheral mechanisms of CTTH. In 40 patients with CTTH and 40 healthy controls, we used electrical stimulation to determine sensory threshold (SPT) and pain perception threshold (PPT) and the characteristics of the electrophysiological sensory nerve action potential (SNAP): initial sensory response (ISR) and supramaximal response (SMR). We then calculated the intensity differences between thresholds (IDT), namely SPT-PPT, ISR-SMR and SMR-PPT, and correlated these IDTs with psychological characteristics: trait and state anxiety, depression, and emotional regulation. The SPT, together with the ISR and SMR thresholds, were higher (*p* < 0.01) in CTTH patients. The SMR-PPT IDT was smaller and correlated with significantly higher indicators of depression, state and trait anxiety, and poorer cognitive reappraisal. CTTH patients have less capacity to recognize non-nociceptive sensory stimuli, greater tendency toward pain facilitation, and a poor central pain control requiring higher stimulation intensity thresholds to reach the start and the peak amplitude of the SNAP. This is consistent with relative hypoexcitability of the Aβ nerve fibers in distant regions from the site of pain, and therefore, it could be considered a generalized dysfunction with a focal expression. Pain facilitation is directly associated with psychological comorbidity.

## 1. Introduction

Tension-type headache is the most frequent form of headache and a hard-to-treat disease with an estimated prevalence of between 30% and 78% throughout the lifetime of the people who suffer it [1], with an estimated incidence of chronic tension-type headache (CTTH) of between 2% and 3% in the general population [2] that causes considerable functional limitations and have significant personal and economic repercussions [3,4].

**Figure 1:**
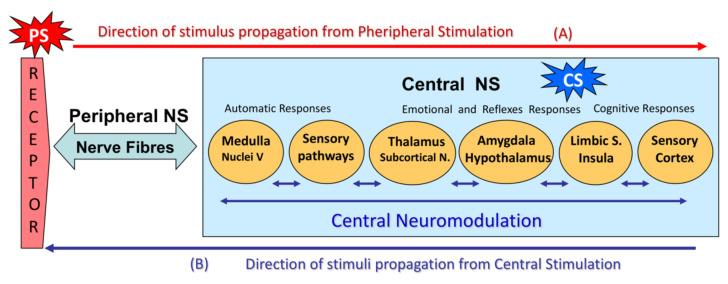
Diagram showing the physiology of craniofacial sensitivity, sensory perception of pain, and pain modulation.

The pathophysiological mechanisms of CTTH have yet to be fully defined but are thought to be due both to hypersensitivity in pericranial structures and local nerve receptors [5,6,7,8] and deregulation or hypersensitization in central nervous system (CNS) pain modulation pathways [6,7,8,9,10]. These alterations may be interlinked in a self-activating loop that perpetuates cranial pain [11,12,13] (Figure 1).

As show in Figure 1, sensory perception is a central nervous system process that can be activated by (A) a stimulus that activates receptors or peripheral nerves (peripheral generator) or (B) a nerve impulse initiated in central nerve system circuits (central generator).

(A) A peripheral stimulus (PS) activates external (skin) or internal (fascia, muscles, viscera) neuroreceptors that stimulate nerve fibers. Large-diameter, myelinated nerve fibers (Aα and Aβ) are more easily excitable than small nerve fibers (Aδ and C).
  The nerve fibers carry the stimulus to the CNS. Large-diameter, myelinated nerve fibers (Aβ) conduct signals more rapidly than small nerve fibers (Aδ and C).
  The first stimuli to reach the spinal cord come from large-diameter, myelinated nerve fibers, which then control the input from the remaining fibers in the dorsal horn of the spinal cord (“gate control”).
  If signals from the small fibers are particularly intense (intense stimulus) and/or selective, they will override the gate-control mechanism.
  Once in the spinal cord (CNS), the nerve impulses travel along the lemniscal pathways or posterior funiculi to suprasegmental structures.
  In the head and neck, sensory stimuli travel directly to the trigeminal sensory nucleus in the brain stem.
  The impulses reach subcortical structures (thalamus, subcortical nuclei) and then the amygdala and hypothalamus, triggering the emotional reaction and its vegetative response, which in turn is a sensory modulation mechanism.
  From the thalamus, impulses travel to cortical structures, such as the limbic cortex, insula cortex, and somatosensory association cortex, generating the sensory cognitive response that perceives the sensation. The sensation is in turn appraised and linked to a prior experience, and this may generate a conditioned response
  All these CNS structures are interrelated and constitute the central neuromodulation mechanism.
(B) The circuit can be reversed: an impulse (central stimulus, CS) can be generated primarily in sensory-related CNS structures (central sensory nuclei, psycho-emotional regulation circuits, sensory cortex, etc.), which will trigger perception.
  Central hyperstimulation due to functional alterations in pain perception circuits would lead not only to an abnormal, exaggerated perception of pain but also to peripheral tissue hypersensitivity. This stimulates nerve fibers (peripheral hypersensitization), which in turn feed back to the CNS to generate secondary central hypersensitization.
  Both mechanisms can be found in chronic pain processes, making it difficult to define the origin of the self-activation loop [13,14,15].

Stimuli can reach the peripheral nervous system (PNS) from outside (exteroception) or inside (interoception) the body. Excitability is the physiological capacity of the membrane of a nerve fiber to generate an action potential when stimulated. Large-diameter, myelinated nerve fibers (Aα and Aβ) are more excitable and transmit nerve impulses more rapidly than small and unmyelinated fibers (Aδ and C) [16,17]. The excitability of the membrane of a nerve fiber can depend on the intensity and frequency of the stimuli it receives so that a preceding stimulus can cause a transient state of initial hypoexcitability that transitions to hyperexcitability. This peripheral modulation of axonal membrane excitability is more accentuated in large-diameter, myelinated nerve fibers [14,18].

The CNS may be stimulated extrinsically from peripheral nerves or intrinsically by stimuli generated in the CNS itself. Depending on the quality of the stimulus and the type of synapse involved, this will either activate or inhibit the nerve pathways, circuits, and neural networks and modulate CNS activity. The processing of nerve impulses travelling along the spinal cord, brain stem, and brain leads to the perception and evaluation of sensation, setting in motion certain responses that can be reflexive, automatic, or voluntary and are either conditioned or unconditioned or unconscious or conscious [19,20] (Figure 1).

During processing, the sensory impulse leaves an “imprint” or sensory memory that can be accompanied by an emotional component [5,21]. Responses to successive exposure to the same stimulus will be influenced by this sensory and emotional imprint and can facilitate or inhibit the stimulus. This is the process of central sensitization, in which hypersensitization is determined by facilitation and hyposensitization by inhibition. This process is not limited to physical and/or biochemical functional changes in the CNS but can also modulate excitability in peripheral nerve fibers [5,6,15,22,23,24,25].

Sensitization, therefore, can be generated from external or internal sensory impulses in the neural circuits involved in the sensory and/or emotional regulation of pain, thus activating a sensation or feeling with a sensitive sensory response that is the same or similar to the response that would have been elicited by an external stimulus [5,6,21,26]. This is consistent with the notion of pain as a sensory and emotional experience “associated with actual or potential tissue damage” [27].

We hypothesized that CTTH may involve primary functional alterations in the central pain modulation circuits that facilitate the perception of pain, promoting in turn peripheral hypersensitivity that causes changes in neuronal excitability and leads to and enhances central hypersensitization. To test this hypothesis, we applied electrical stimulation to healthy volunteers and patients with CTTH to elicit subjective non-nociceptive pain responses and objective responses related to neuronal excitability. We correlated these with various psychological parameters, including anxiety, depression, and emotional regulation, in order to identify probable differences and associations with psychopathological comorbidity.

## 2. Materials and Methods

### 2.1. Participants

Forty subjects with a diagnosis of CTTH (age range 30–65 years, median 50.35, SD 10.12) and another forty healthy controls (HC) with no headache (age range 23–59 years, median 40.65, SD 10.51) were included in the study. Subjects with CTTH were recruited from the Neurology Department of the Virgen de la Victoria University Hospital in Malaga (Spain). The study was approved by the Ethics Committee of the University of Malaga. All subjects participated voluntarily and signed an informed consent form before inclusion. This study complies with the ethical criteria defined in the Declaration of Helsinki of 2014 and Organic Act March 2018, of 5 December, on the Protection of Personal Data and Guarantee of Digital Rights.

The diagnosis was made by a neurologist specialized in headache, following the criteria of the International Classification of Headache Disorders [1]. The electrophysiology study was performed by a specialist in clinical neurophysiology. Psychometric data were collected by a clinical neuropsychologist. All raters were blinded to the results of the other investigators. The data were analyzed by an independent evaluator.

The inclusion criteria for subjects with CTTH and healthy controls were: aged between 20–65 years and no psychotropic drugs or analgesics taken in the 72 h prior to the study. Individuals diagnosed with a CNS or PNS disorder or who might present technical skin and/or subcutaneous tissue issues that could make it difficult to stimulate or register sensory responses were excluded [28]. Patients with more than one type of headache (such as chronic tension-type headache and migraine) were not included.

### 2.2. Materials

#### 2.2.1. Electrophysiology Study

The electrophysiology study was performed with a Sierra Wave electromyography system, version 7 (Cadwell, WA, USA), with integrated electrical neurostimulator and disposable surface electrodes and dermal temperature probe from the same brand.

#### 2.2.2. Questionnaires

**Figure 2:**
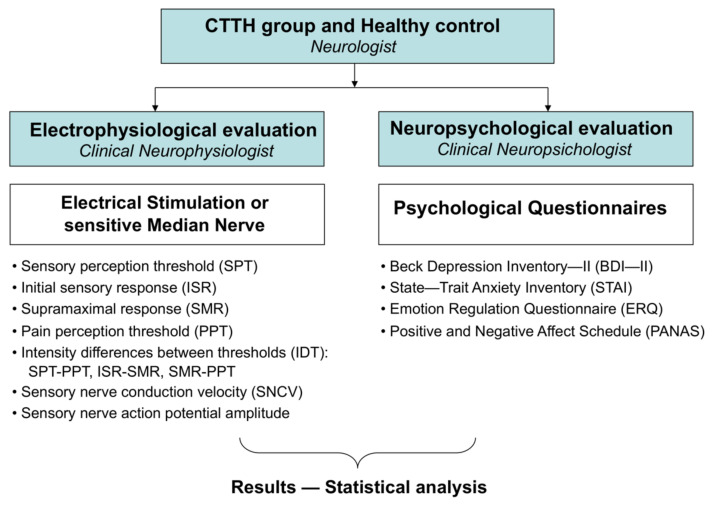
Methodological scheme.

The following questionnaires (Figure 2) were used to collect psychological variables:

Beck Depression Inventory—II (BDI—II) to determine the existence and severity of depression symptoms [29,30];
State—Trait Anxiety Inventory (STAI) to evaluate anxiety as a temporary state (state anxiety) or as a personal characteristic (trait anxiety) [31,32];
Emotion Regulation Questionnaire (ERQ) to separately assess cognitive reappraisal or regulation prior to an emotional experience and expressive suppression after an emotional experience [33,34];
Positive and Negative Affect Schedule (PANAS) to evaluate the subject’s emotional recognition, as a positive or negative affect, either as a trait or a state (trait or state positive affect, trait or state negative affect) [35,36].

### 2.3. Methods

Antidromic stimulation of the right median nerve was performed by applying an electrical stimulus to the anterior flexure of the wrist and collecting the sensory response in the second finger of the hand. The position of the recording, reference, ground, and stimulation electrodes remained unchanged throughout the procedure in order to achieve a reliable and reproducible recording [12,16,37,38,39].

The characteristics of the study and the subjective parameters to be collected were explained to all subjects. The following subjective responses reported by the subject were evaluated: the sensory perception threshold (SPT) or intensity at which the subject first recognized the applied stimulus and the pain perception threshold (PPT) or intensity at which the subject begins to recognize the stimulus as painful. The following objective responses observed during electrophysiology tests were evaluated: initial sensory response (ISR) or intensity at which the start of a detectable SNAP is observed and supramaximal response (SMR) or intensity at which the SNAP reaches its peak amplitude (Figure 2).

The intensity differences between thresholds (IDT) were determined in the following parameters: SPT-PPT, ISR-SMR, and SMR-PPT (Figure 2).

The electroneurographic parameters sensory nerve conduction velocity (SNCV) and SNAP amplitude were measured in m/s and μV, respectively, to determine normality according to the reference values (Figure 2).

The study was performed with the subject at rest, lying on an examination table (in position at least 15 min before the examination). The room temperature was maintained at 23–25 °C, and the skin temperature, measured with a temperature probe held by the patient, was between 30 and 32 °C. The electrical stimulus consisted of a quadrangular pulse lasting 0.1 ms, which was increased in increments of 1 mA until the perception threshold was reached and the initial SNAP obtained, after which it was increased in 2 mA increments until the remaining thresholds and responses had been obtained. The forced choice method was used to correctly measure the SPT and PPT, increasing and decreasing the intensity on at least 3 occasions to pinpoint the exact intensity generated by the sensation evaluated [40,41]. To determine ISR and SMR, 5 µV gains per division were used. ISR was defined as the minimum intensity needed to obtain a detectable SNAP without amplification. SMR was defined as the intensity needed to obtain an SNAP whose amplitude did not increase after applying a stimulus that was 20% more intense than the threshold. The stimuli were applied at intervals of between 25 and 55 s to avoid the expectation phenomenon [16].

### 2.4. Statistical Analysis

Data from the experimental and control groups were analyzed descriptively. The quantitative, psychological, and electrophysiological variables were expressed as mean and standard deviation (SD). The Kolmogorov–Smirnov normality test was used to test the normality of the distribution of quantitative data, and the Levene test was used to verify the equality of variances of all levels of each factor. Following this, the psychological and electrophysiological variables from both groups (CTTH and HC) were compared using the independent samples *t*-test in the case of normally distributed quantitative variables and the Mann–Whitney U test in the case of non-normally distributed variables.

Pearson’s linear correlation coefficient was used to test for linear correlations between electrophysiological variables in the experimental and control groups separately. The linear correlation coefficients between psychological and electrophysiological variables were also calculated in each group.

Logistic regression analysis was performed to determine the capacity of the most sensitive electrophysiological variables to classify the data. The default variable input method was used. The effect was controlled by age and gender, taking age and sex as independent variables and SPT, ISR, and SMR as dependent variables and analyzing the relationship between each variable using the Pearson chi-squared goodness-of-fit test.

The diagnostic accuracy of electrophysiological variables that differed significantly between the HC and CTTH groups was evaluated with Receiver Operating Characteristic (ROC) curves, using cut-off points with the highest specificity and sensitivity.

Statistical analysis was performed on IBM SPPS Statistics v.27 (IBM, Armonk, NY, USA). A 95% confidence interval was used in all tests, and significance was set at *p* < 0.05.

## 3. Results

### 3.1. Differences in Subjective and Objective Electrophysiological Responses between Subjects with Chronic Tension-Type Headache and Healthy Controls

**Figure 3:**
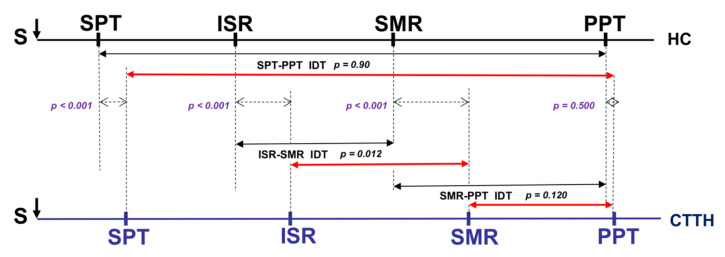
Comparison of sensory responses to electrical stimuli in CTTH patients and healthy controls. CTTH, chronic tension-type headache; HC, healthy control; IDT, intensity difference between thresholds; ISR, initial sensory response; PPT, pain perception threshold; S, stimulus; SMR, supramaximal response; SPT, sensory perception threshold.

**Figure 4:**
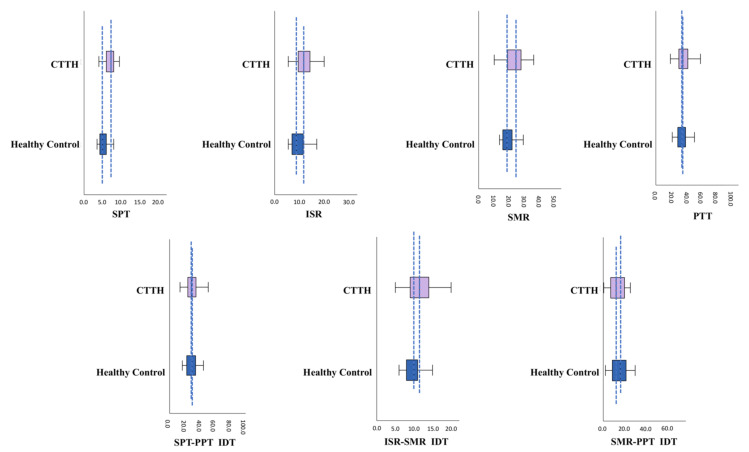
Box plots comparing subjective and objective electrophysiological responses and IDTs between healthy controls and CTTH patients. IDT, intensity difference between thresholds; ISR, initial sensory response; PPT, pain perception threshold; SMR, supramaximal response; SPT, sensory perception threshold.

Table 1 and Figure 3 and Figure 4 compare the stimulus intensity values for the defined response thresholds and the intensity values between the IDTs analyzed. Significant differences were observed between groups (HC and CTTH) in the subjective SPT response (*p* < 0.001) and the electrophysiological ISR (*p* < 0.001) and SMR (*p* < 0.001) responses, all of which were higher in the CTTH group vs. HCs. No differences were found between groups with respect to the subjective PPT response (*p* = 0.372). The only IDT in which a significant difference was observed was ISR-SMR (*p* < 0.001), which was greater in CTTH subjects. The SPT-PPT IDT (*p* = 0.090) and the SMR-PPT IDT (*p* = 0.302) did not differ between groups, and there were no significant differences in the electrophysiological SNCV parameters (*p* = 0.526) or SNAP amplitude (*p* = 0.613).

Table 2 shows the correlations between subjective SPT and PPT responses, electrophysiological ISR and SMR responses, and IDTs between the CTTH patients and HCs.

The logistic regression study showed that the statistically significant differences observed between groups in SPT, ISR, and SMR are independent of the age and gender effect (X^2^ = 10.276, *p* = 0.246), thus proving the goodness-of-fit null hypothesis and showing that the model is capable of correctly classifying 80% of the subjects.

**Figure 5:**
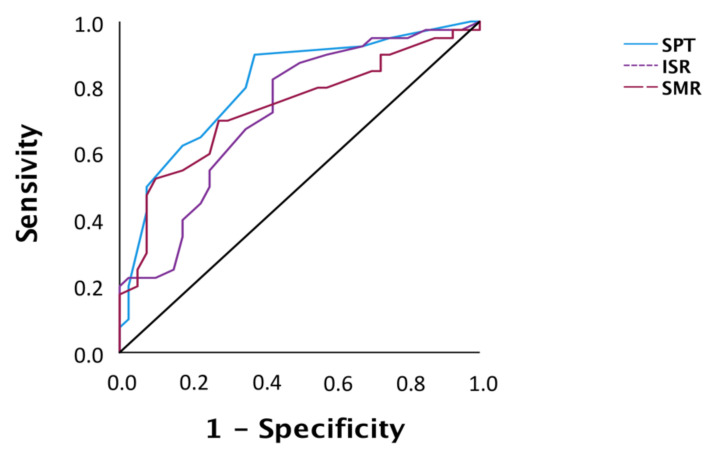
ROC curve of subjects with or without CTTH.

The ROC curve showed that the SPT (90% sensitivity and 63% specificity), ISR (82.5% sensitivity and 58% specificity), and SMR (70% sensitivity and 72.5% specificity) responses were diagnostically accurate (Figure 5).

### 3.2. Psychological Differences between Subjects with Chronic Tension-Type Headache and Healthy Controls

Table 3 shows the differences in psychological questionnaire scores between the CTTH group and HCs. In HCs, these scores were within reference limits for the healthy population. Scores for state anxiety (*p* < 0.001), trait anxiety (*p* < 0.001), depression (*p* < 0.001), and state negative affect (*p* < 0.001) were significantly higher in the CTTH group vs. HCs, while score for state positive affect (*p* < 0.001) and trait positive affect (*p* = 0.020) and cognitive reappraisal (*p* < 0.005) were significantly lower in the CTTH group vs. HCs.

### 3.3. Correlations between Electrophysiological and Psychological Variables

In the control group, a positive correlation was observed between trait positive affect and PPT (r = 0.338, *p* = 0.033) and also between PPT-related intervals: SPT-PPT (r = 0.344, *p* = 0.030) and SMR-PPT (r = 0.379, *p* = 0.016).

**Figure 6:**
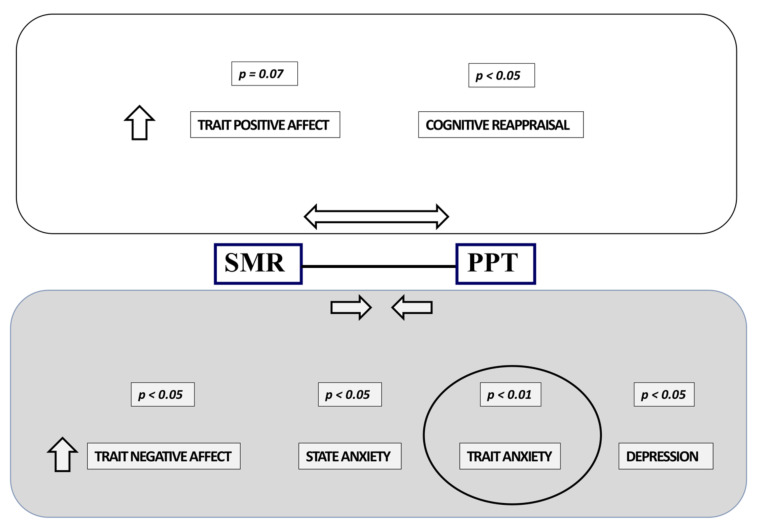
Correlation between pain facilitation and neuropsychological variables.

In the CTTH group, a positive correlation was observed between PPT and the psychological variable trait positive affect (r = 0.306, *p* = 0.055) and a negative correlation between the SMR-PPT interval and the psychological variables trait negative affect (r = −0.315, *p* = 0.047), state anxiety (r = −0.360, *p* = 0.022), trait anxiety (r = −0.431, *p* = 0.005), and depression (r = −0.368, *p* = 0.019). The SMR-PPT interval only presented a significant positive correlation with respect to cognitive reappraisal (r = 0.324, *p* = 0.042) (Figure 6).

## 4. Discussion

The SPT indicates the degree of subjective non-nociceptive sensory discrimination. Physiologically, it is determined by the activation of large-diameter, myelinated nerve fibers (Aα and Aβ) that are more susceptible to excitation and that rapidly transmit the impulse to the CNS, where it is initially recognized (Figure 1) [10,12,15].

In our study, the SPT was significantly higher in patients with CTTH compared to healthy controls (Figure 3 and Figure 4). In patients with CTTH, this has been previously observed in both trigger points [42,43] and other body areas [44,45,46] and could be due to a lower capacity for subjective sensory discrimination due to an alertness/attention deficit in the CNS (central dysmodulation) or hypoexcitability in Aα and Aβ nerve fibers, which are more excitable and conduct nerve impulse more rapidly (peripheral dysmodulation) [40].

The ISR and SMR are objective parameters related to nerve excitability. Both responses are recorded by stimulating Aβ fibers. The ISR objectively indicates the activation of a sufficient number Aβ sensory nerve fibers to evoke a sensory potential capable of being detected in the electrophysiological study, while the SMR indicates the activation of all the sensory fibers in the nerve. Although small nerve fibers (Aδ and C), which are associated with thermal and pain sensitivity, can be activated at the SMR intensity, they do not contribute to the SNAP observed in conventional electrophysiology [47].

The stimulus intensity required to reach the ISR and SMR was higher in subjects with CTTH compared to HCs, which suggests that Aβ nerve fibers in subjects with CTTH are less susceptible to excitability compared to healthy individuals (Figure 3 and Figure 4). Studies have shown that hyperstimulation of a nerve can determine the hypoexcitability of its fibers, with the larger, myelinated Aβ nerves being the most easily modulated [5,14,18,40].

We were unable to observe the degree of excitability of Aδ and C fibers since they are not expressed in SNAPs. However, we, like other authors, have assumed that they are either not hypoexcited or less hypoexcited than large-diameter fibers [14,18,40].

The hypoexcitability of Aβ fibers was observed at a point distant from trigger points that are potentially hypersensitive in patients with CTTH, suggesting that it may be a diffuse event. We evaluated this finding in the median sensitive nerve because of its higher sensibility and stability response recording; the evaluation of this sensitive response in other sensitive nerve in lower limbs may be of interest in other new studies.

When the intensity of the electrical stimulus is increased, a PPT is reached, in which the sensory interpretation changes from non-nociceptive to painful. The PPT is an indicator of the capacity to recognize and modulate pain perception on a psychosensorial level and is determined by activation of the Aδ and C fibers (already achieved by delivering the intensity needed to achieve an SMR) and by the successive steps, connections, and regulations that occur from the time the painful sensory impulse enters the CNS until it reaches the somatosensory perceptive and associative cortex (Figure 1).

Other authors [46,48] have also failed to observe any differences in PPT between subjects with CTTH and healthy controls, and this has also been reported in other types of patients with idiopathic pain symptoms, such as fibromyalgia or local regional pain syndrome [49,50,51]. This, however, is a controversial finding since other authors contend that patients with CTTH have a lower PPT [52,53,54,55,56].

We attempted to resolve this issue by evaluating the SMR-PPT IDT. This interval indicates the intensity increase required from excitation of all the sensory fibers of the nerve until pain is perceived; we have therefore called it the “pain permeability interval”. Although the differences observed were not significant, this interval is shorter in subjects with CTTH compared to healthy controls (Figure 3 and Figure 4), leading us to believe that an alteration in central pain regulation circuits facilitates central pain perception.

If there is indeed a central, generalized pain facilitation mechanism, we need to consider why cranial pain in patients with CTTH is localized instead of generalized as it is in fibromyalgia. Although this is a questionable assumption, one possible explanation is that the frontotemporal cranial structures, which receive their sensory innervation from the trigeminal nerve, have more direct access to the CNS and less input modulation than in body areas where access is through the spinal cord gate-control filter [13,42,43,57,58].

According to the central sensitization theory of chronic pain, competent nociceptive stimuli can trigger neuroplasticity processes in the central circuits that transmit, modulate, and perceive pain; thus, the perception of a particular pain is either facilitated and perpetuated permanently or elicited with a far lower intensity pain stimulus [5,6]. This theory is based on the hypothesis that external nociceptive stimuli are the primary drivers of these changes due to myofascial contraction or alteration and local biochemical and inflammatory changes. This leads to secondary hypersensitization in central circuits, which in turn triggers peripheral adaptation responses in sensory receptors and local pericranial myofascial territories (trigger points) that cause and perpetuate the situation [5,6,22,52,56,59,60,61]. In our study, we found that subjects with CTTH presented a significantly lower permeability for pain, so we believe that the primary cause for hypersensitization is central dysmodulation.

On a neuropsychological level, we found significantly higher rates of state anxiety and trait anxiety, significantly higher rates of depression, and a far lower capacity for emotional regulation in patients with CTTH compared to healthy controls (Table 3). These neuropsychological alterations are closely related to their extremely short “pain permeability interval” (Figure 3 and Figure 4), leading us to believe that central dysmodulation mechanisms together with neuropsychological alterations play an important role in the origin of pain perception facilitation in subjects with CTTH.

We did not find any correlation between the higher SPT observed in subjects with CTTH and any particular psychopathological or emotional regulation trait, which suggests to us that this lower discrimination sensitivity to sensory perception is an independent defining trait of the psychological variables found in subjects with CTTH and that this could be related to attention span or sensory avoidance in these subjects, a hypothesis that could be explored in subsequent studies.

Studies have shown that the CNS acts as the primary sensory trigger in circuits that transmit, modulate, perceive and evaluate feelings and that these can precede the functional and structural modifications that occur as an organic expression of such feelings in peripheral areas, where, by a process of sustained activation (such as gestures, postures, muscle, or myofascial tone), they bring about functional and structural modifications that may in turn act as the causal mechanisms of a process of hypersensitization and pain re-entry facilitation [15,20,21,26].

Some authors have observed that people with chronic pain, specifically patients with CTTH, present a greater psychopathological burden in the form of anxiety, depression, or emotional management difficulties [10,15,62,63,64]. These are frequently linked to other comorbidities that cause chronic pain, such as fibromyalgia, osteoarticular pain, oral and facial pain, abdominal pain, postoperative pain, or neurovegetative alterations, such as irritable bowel syndrome, tachycardia, etc. [5,6,15,63].

Neuropsychological alterations may be secondary to or influenced by a persistent painful life experience although their primary or secondary link to CTTH is controversial and should be explored in future studies. However, it is evident that their presence worsens and/or perpetuates CTTH symptoms [15].

To summarize, CTTH is associated with primary central pain facilitation that can set in motion by a particular painful experience that can then lead to hypersensitivity in peripheral tissue structures. These alterations lead to sensory hyperstimulation that may induce a relative hypoexcitability of Aβ fibers and further facilitates the input of pain sensations in the CNS, mediated peripherally by Aδ and C fibers. This re-entry loop perpetuates the painful experience and reinforces central hypersensitization. The close correlation with psychopathological alterations, such as anxiety, depression, or lack of emotional control, can be an expression of the cause itself or a consequence of the sustained painful life experience, thus helping to perpetuate and reinforce the pain [64,65,66,67,68].

Despite the fact that the diagnosis of CTTH is clinical, it would be necessary to evaluate these patients in both neurophysiological and neuropsychological aspects to better define the profile of each one and adapt the best therapeutic management to avoid the perpetuation and reinforce sensitization.

## 5. Conclusions

The nervous system is an integrated, dynamic macro-complex in which all functions are interdependent, synchronized, and reciprocal and modulated by plasticity mechanisms. Therefore, chronic processes can be difficult to isolate or disassociate from a physiological perspective. This study helps show that the primary cause of pain perception dysmodulation in patients with CTTH might be a primary predisposition in the CNS, which leads to secondary peripheral hypersensitization and hypoexcitability of Aβ fibers. Greater pain facilitation is closely associated with a greater psychological comorbidity burden of anxiety, depression, and emotional disturbance. We believe that our findings can improve the conceptual understanding of CTTH and help clinicians achieve a more effective and sustained therapeutic response.

## Figures and Tables

**Table 1:** Electrical stimuli intensity response thresholds and intensity difference between thresholds in CTTH (*n* = 40) and healthy controls (*n* = 40).

	Healthy Controls (in mA)		CTTH (in mA)		*p*
	MD ± SD	Median	MD ± SD	Median	
SPT **	5.38 ± 1.34	5.00	7.49 ± 2.45	7.25	<0.001 ^b^
ISR **	9.70 ± 3.33	8.75	13.19 ± 5.15	11.75	<0.001 ^a^
SMR **	19.66 ± 4.08	19.00	24.93 ± 3.33	25.00	<0.001 ^b^
PPT	36.65 ± 10.92	35.50	39.24 ± 14.58	36.50	0.372 ^a^
SPT-PPT IDT	31.28 ± 10.94	30.00	31.75 ± 13.36	29.25	0.090 ^a^
ISR-SMR IDT **	9.96 ± 2.11	10.00	11.74 ± 3.59	11.50	<0.01 ^b^
SMR-PPT IDT	16.99 ± 10.93	16.00	14.31 ± 12.08	12.00	0.302 ^a^

Quantitative variables are expressed as mean ± standard deviation and median. CTTH, chronic tension-type headache; IDT, intensity difference between thresholds; ISR, initial sensory response; PPT, pain perception threshold; SMR, supramaximal response; SPT, sensory perception threshold. ^a^ *t*-test, ^b^ Mann–Whitney U-test. ** *p* < 0.01.

**Table 2:** Correlation between subjective and objective electrophysiological responses and IDTs in healthy controls and CTTH.

		ISR	SMR	PPT	SPT-PPT IDT	ISR-SMR IDT	SMR-PPT IDT
SPT	HC	0.602 **	0.644 **				
	CTTH	0.575 **	0.557 **	0.558 **	0.426 **	0.324 **	0.332 *
ISR	HC		0.856 **			0.453 **	
	CTTH		0.897 **	0.418 **	0.351 *	0.415 **	
SMR	HC					0.580 **	
	CTTH			0.562 **	0.511 **	0.774 **	
PPT	HC				0.992 **		0.930 **
	CTTH				0.988 **	0.559 **	0.862 **
SPT-PPT IDT	HC						0.952 **
	CTTH					0.551 **	0.879 **

Data expressed as the Pearson correlation coefficient. CTTH, chronic tension-type headache; HC, healthy controls; IDT, intensity difference between thresholds; ISR, initial sensory response; PPT, pain perception threshold; SMR, supramaximal response; SPT, sensory perception threshold. * *p* < 0.05, ** *p* < 0.01.

**Table 3:** Psychological differences between subjects with CTTH and healthy controls according to their questionnaire scores.

	Healthy Controls (*n* = 40)	CTTH (*n* = 40)	*p*
State anxiety **	20.20 ± 11.90	35.80 ± 14.25	<0.001 ^a^
Trait anxiety **	19.33 ± 9.21	30.90 ± 11.61	<0.001 ^a^
Depression **	7.53 ± 5.42	16.13 ± 9.58	<0.001 ^a^
State positive affect **	31.58 ± 7.19	25.18 ± 7.23	<0.001 ^a^
Trait positive affect *	33.00 ± 6.01	29.25 ± 7.97	0.020 ^a^
State negative affect **	18.08 ± 5.99	25.40 ± 8.70	<0.001 ^a^
Trait negative affect	18.58 ± 5.88	21.35 ± 6.84	0.055 ^a^
Cognitive reappraisal *	4.71 ± 1.38	4.05 ± 1.33	0.033 ^a^
Expressive suppression	3.26 ± 1.42	3.93 ± 1.64	0.055 ^a^

Quantitative variables are expressed as mean ± standard deviation. The data express the numerical score obtained on the questionnaires. CTTH, chronic tension-type headache. ^a^ *t*-test, * *p* < 0.05, ** *p* < 0.01.

## Data Availability

Not applicable.
